# Assessment of hepatitis B surface antigen negative blood units for HBV DNA among replacement blood donors in a hospital based blood bank in Nigeria

**DOI:** 10.4314/ahs.v21i3.22

**Published:** 2021-09

**Authors:** Foluke Atinuke Fasola, Adeola A Fowotade, Adedayo O Faneye

**Affiliations:** 1 Department of Haematology, College of Medicine, University of Ibadan, University College Hospital, Ibadan, Nigeria; 2 Department of Medical microbiology & Parasitology, College of Medicine, University of Ibadan, University College Hospital, Ibadan, Nigeria; 3 Department of Virology, College of Medicine, University of Ibadan, University College Hospital, Ibadan, Nigeria

**Keywords:** Hepatitis B surface Antigen, Hepatitis B Virus, DNA, blood donors, blood safety

## Abstract

**Background:**

Hepatitis B virus infection is one of the greatest threats to blood safety all over the world. The laboratory algorithm based on only the detection of hepatitis B surface antigen (HBsAg) leaves a gap for infected HBsAg negative donors to donate blood during the “window period” (WP) and late stages of infection.

**Objective:**

To estimate the frequency of the presence of HBV deoxyribonucleic acid (DNA) in HBsAg negative blood units screened using two different assays for HBsAg in a high endemic region.

**Methods:**

Frozen serum aliquot of 100 replacement blood donors who donated blood units that were HBsAg negative were retrieved and tested for HBV DNA. Sample positive for HBV DNA was sequenced by Sanger's method, genotyped and the viral load was determined.

**Results:**

One sample (1%) was positive for HBV DNA. The HBV viral load of the sample was 768,000 IU/ml. The partial S-gene of the Hepatitis B virus isolated was genotype E using the NCBI viral genotyping tool.

**Conclusions:**

There is still a risk of HBV infected blood unit escaping detection when donor testing is limited to HBsAg screening. The use of NAT which can substantially reduce HBV infected blood donors from the donor pool should be considered.

## Background

For the supply of safe blood, the major strategy recommended by World Health Organization (WHO) is the collection of blood from low risk voluntary non-remunerated blood donors. As against the WHO recommendation, voluntary blood donors constitute less than 20% of the blood donors in Nigeria thereby leaving the transfusion service to depend heavily on replacement and sometimes paid blood donors to make blood available to recipients.^1^ The blood donor eligibility is strongly dependent on the result of the laboratory screening test. The prevalence of transfusion transmitted infections in replacement blood donors are argued to be comparable to that in voluntary blood donors if both are first time donors.^2^ But because replacement blood donors are often times first time donors, they have a higher risk of transfusion-transmitted viruses (TTV) than voluntary blood donors who are likely repeat donors. Among the TTVs, similar to findings in other countries, hepatitis B virus (HBV) is the most prevalent with the highest residual risk of transmission to blood recipients.^3^,^4^

The burden of HBV in Sub-Saharan Africa (SSA) is high with highest concentration in West Africa where Nigeria is located.^5^ The seropositivity rate for hepatitis B surface antigen (HBsAg) among blood donors in Nigeria is between 8 and 22%.^3^,^6^,^5^ Blood donor occult HBV infection ranges from 10.6% to 17% in Nigeria.^5^ Infection by hepatitis B virus can be complicated by liver cirrhosis and liver cancer without the potential for a cure. In 2015, HBV infection was responsible for an estimated death of 887 000 people worldwide.^6^ The identification of HBV carrier status in blood donors is critical to eliminating the transmission of HBV through blood transfusion. The estimated transfusion risk models for HBV exceeds that of HIV in Africa.^5^

Blood donor screening for HBV infection in Nigeria started in the late 1980s when only rapid test which detected HBV surface antigen (HBsAg) by immunochromatography was carried out by a few blood banks. Despite the use of “immunochromatography test strip only” screening tests, cases of post-transfusion viral hepatitis were still being reported.^7^ This informed migration to the use of Enzyme-Linked Immunoassay (ELISA), to detect HBsAg, which is now in use for almost two decades in Nigeria, to screen blood donors for HBV. One of the shortcomings identified with using ELISA is variation in the analytical performance of different assay relating to the window period of the infection.. Window period for serology test is 59 days^8^ during which the virus is transmissible. Given this long window period, most developed countries have included HBV nucleic acid testing (NAT) in the laboratory screening of donors. NAT would identify contaminated blood from donors in the window period of the disease. In addition to the risk of transmission of HBV during the window period, transmission could occur through donors with occult HBV infection. Occult HBV infection (OBI) in donors is characterized by the presence of HBV DNA in the liver and fluctuating low level of viremia not detected by current HBsAg tests. Occult HBV in a blood donor represents a potential source of transfusion-transmitted HBV. OBI is observed during the incubation period of acute infections, the tail-end stage of chronic hepatitis B, low-level viral replication after recovery from hepatitis and infection with escape mutants.^9^ Since nucleic acid test (NAT) is considered the gold standard test in HBV infection identification, this study was carried out using NAT. This pilot study aims to estimate the frequency of the presence of HBV DNA in hepatitis B surface antigen (HBsAg) negative blood units screened using two different assays types in the context of a flexible blood donor evaluation.

## Methods

### Study location and population

The study was carried out at the University College Hospital (UCH) Blood Bank, Ibadan. The UCH is a tertiary health institution located in Oyo state, South Western Nigeria which was established in 1957 with the aim of Research, Training and provision of Service. The hospital-based blood bank collects blood from an average of 600 donors in a month when working in full capacity. However, during the period of one month June to July 2019 when the study was conducted 450 blood donors were seen in the blood bank. The blood bank recruits predominantly replacement blood donors with some voluntary blood donors. Routinely the blood donors are screened with rapid kits for HBV, hepatitis C virus (HCV), and human immunodeficiency virus (HIV). Those who were non-reactive to tests for the viruses are then bled. Those who are reactive are permanently deferred as well as referred to appropriate clinic for treatment. The blood units of the non-reactive donors are further subjected to ELISA test for HBV, HCV and HIV as well as syphilis. The blood units that are also non-reactive in this second test are the ones released for patient care. The blood bank collects (whole blood), prepares red cell concentrate, fresh frozen plasma, cryoprecipitate and platelet concentrate and stores blood and blood products for the use of the patients managed in the hospital. The donor population recruited in this study were replacement blood donors who presented to the donor section of the blood bank.

The sample size for the study was estimated using the formulae by Schaeffer RL *et al*^10^

Belmont, California 1990 based on 8.9% prevalence rate according to the study reported by.^11^ Using the formula for a population of 450 blood donors who attended the blood bank in the month of study, the sample size was 98 with a 95% confidence limit,

### Study design and sample collection

This was a cross sectional pilot study carried out in the hospital-based blood bank. Ethical approval for the study was obtained from the joint University of Ibadan/University College Hospital, Ibadan (UI/UCH) ethical committee. Six milliliters of venous blood sample was collected from 300 replacement blood donors who had been certified fit to donate at the hospital blood bank. The blood was allowed to clot and serum was decanted into 2 aliquots and immediately frozen at -80°C until analysis. Three hundred blood donors were recruited in order to have adequate number of blood donors that will be negative for HBsAg while also being negative and non-reactive to other transfusion transmitted infections screened for in the blood bank. The demographic characteristics of the seronegative donors were recorded. All the donors went through the routine donor evaluation procedure of the blood bank.

The routine evaluation procedure included verbal questioning on health and lifestyle, anaemia screening to determine fitness and rapid screening tests for HBV, HCV and (HIV). Donor questions specific to HBV risk included: sexualctivity, intravenous drug abuse, sharing of sharp objects, tattoo, scarification and tribal marks, history of jaundice, previous diagnosis of HBV infection and blood transfusion. The blood samples from the donors, according to the tradition of the blood bank was further screened for syphilis, HBV, HCV and HIV with semi-automated conventional enzyme-linked immunosorbent assay (ELISA) tests by the blood bank. The ELISA test kit for HBV was Monolisa™ HBsAg ULTRA ELISA kits manufactured by BIORAD (3, bd Raymond Poincare, 92430 Marnes-la-Coquette-France) a qualitative one-step enzyme immunoassay technique of sandwich-type for the surface antigen of HBV (HBsAg) in the serum. The kit uses monoclonal and polyclonal antibodies selected for their ability to bind themselves to the various subtypes of HBsAg. Diagnostic specificity and sensitivity for the Monolisa kit were 99.94 and 100% respectively. The rapid and ELISA test kits used for the study were same as that used by the blood bank. The frozen aliquot from consecutive 100 blood donors whose blood units were HBsAg, anti - HIV, syphilis and, anti-HCV serologically negative then underwent HBV DNA detection and viral load by polymerase chain reaction analysis. The 100 were selected on the basis of being the first 100 donors out of the 300 donors whose samples were seronegative for all TTI tested.

### HBV DNA Detection

Total DNA was extracted from the first 100 serum samples that tested negative for HBsAg using in-house DNA extraction protocol described by Wang *et al.*^12^ The extracted DNA were quantified using NanoDrop spectrophotomer and extracts with concentration above 20ng/ul were used for PCR. The DNA were then tested for HBV DNA using primers that targets the Pre-S gene of the virus. The Pre-S gene of HBV was amplified in a semi-nested PCR protocol. The firstround PCR was performed in a 25µl reaction containing 5µl of red load PCR mix by Jena Biosciences (Jena Germany), and 2µl eachof primers 979 (5′CAAAAGACCCACAATTCTTTGACATACTTTCCAA3′) and SF (5′GTGTCTTGGCCAAAATTCGCAGT3′) with 5µl of DNA and 11µl of nuclease-free water. The amplification was done in ABS 7000 thermal cycler following an initial denaturation of 95°C for 5 minutes, followed by 35 cycles of 95°C for 30 seconds, 62°C for 45 secs and 72°C for 45 seconds and a final elongation of 72°C for 10 minutes. The amplified product of the first-round PCR was used as a template for the second round PCR in a similar 25µl reaction volume using primers 979 and MC-F (5′TCGGATCCGGTATGTTGCCCGTTTGTC3′ and the same cycling condition. The amplified product was detected using agarose gel electrophoresis with 2% agarose and TBE used as running buffer, the product was then visualized in LED transilluminator.

### HBV DNA Viral load

The HBV DNA viral load of the positive sample was quantified by Bio-Rad I-Cycler using HBV viral load kit by EliGene (Elisabeth Parmacon LTD Check Republic) according to the manufacturer's instruction. The detection limit of this assay is 1 IU/ml HBV DNA viral load

### HBV Genotyping

The positive PCR product was purified using a commercially available PCR purification kit by Jena Biosciences (Jena, Germany) according to the manufacturer's instruction and sequenced in both directions using primers Mac-F and 979 on an ABI Prism 3130xl (Applied Biosystems) genetic analyser. A consensus sequence was generated for the sequences isolate using CLC Main Workbench 7.6.2 software (CLC bio, Cambridge, MA, USA). The sequence data were edited using Bio-Edit software and the Nucleotide BLAST search (http://www.ncbi.nih.gov/BLAST) was used to acquire homologous sequences. Multiple sequence alignment of the study sequence and others from GenBank was done using the MAFFT alignment tool (https://mafft.cbrc.jp/alignment/server/). Phylogenetic analysis was done using MEGA 5.05 software.^11^ The sequence generated was genotyped using the NCBI viral genotyping tool (http://www.ncbi.nih.gov/projects/genotyping/formpage.cgi)

## Results

Three hundred blood donors were recruited for the study of which 17 (5.6%) were HBsAg positive using rapid kit. Of the 283 that were negative for HBV on rapid test, 38 (13.4%)s were HBsAg positive using ELISA. The age and gender of the first 100 donors whose blood units were seronegative for all TTIs screened were retrieved. The mean age of the donors was 31.3 ± 8.35 years. Most of the participants were males (86.0). More than half of the participants (55.0%) were married and 45% were single. Sixty – six percent (66.0%), 30 % and 4% of the participants had tertiary, secondary and primary education respectively.

Of the one hundred samples, one sample (1%) was positive for HBV DNA.

The sample that was positive for HBV DNA was drawn from a 25-year-old male donor. The donor was syphilis, HIV and HCV negative. The HBV viral load of the sample was 768,000 IU/ml. The partial S-gene of the Hepatitis B virus isolated was successfully amplified, sequenced and determined to be of genotype E using the NCBI viral genotyping tool.

**Figure 1:**
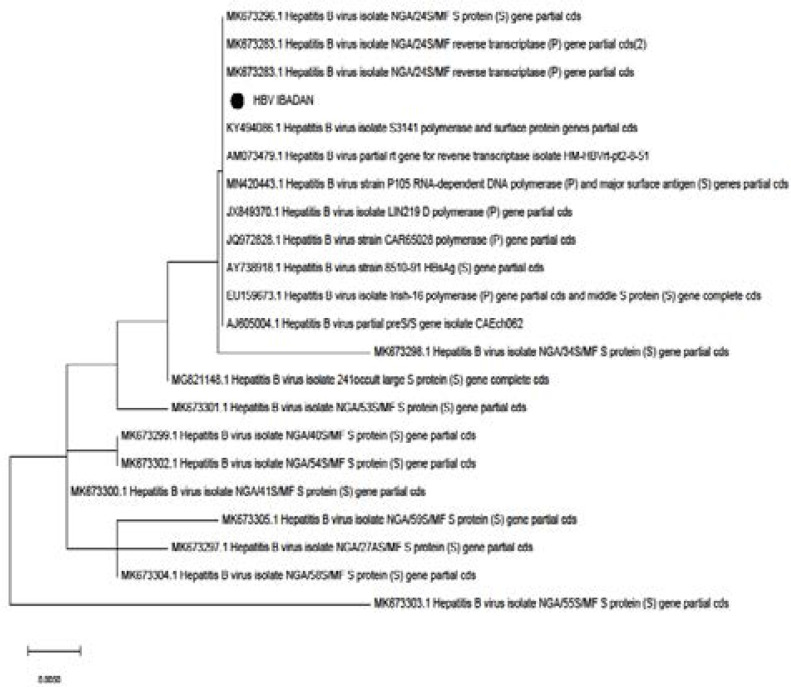
Phylogenetic tree of HBV isolated from this study and others from Nigeria

After the alignment and phylogenetic analysis of the sequences of the isolates in this study with other partial sequences of the Pre-S gene in the GENBANK, the sequence in this study showed the highest similarity with some isolates from Nigeria (Fig. 1).

## Discussion

This pilot study aims to estimate the frequency of the presence of HBV DNA in hepatitis B surface antigen (HBsAg) negative blood units screened using two different assays among replacement blood donors. Our hospital blood bank and some other hospital blood banks in Nigeria continue to use 2 different HBsAg assays: rapid kit and ELISA to screen out blood donors with HBV infection. The effectiveness of this screening algorithm with two complementary tests to prevent HBV infectious units escaping into the pool of blood unit for transfusion is not known. Even though some studies have been conducted on occult HBV infection in blood donors in Nigeria, gap in knowledge still exist. Previous studies carried out HBV nucleic acid test on blood samples that were screened either not in line with the routine practice of the blood bank or studies were conducted using one HBsAg kit assay.^13^, ^14^, ^15^ Therefore, this pilot study was carried out using the screening algorithm of the blood bank and also the two HBsAg kits used by the blood bank to determine real life situation on ground.

The prevalence of NAT positive HBV blood unit in this study was 1%. This suggests that the potential risk of transfusing HBV contaminated blood to the recipient when blood is obtained from replacement donors in a high endemic region is still substantial. The observation of an infectious blood unit escaping detection despite the use of two types of assay of HBsAg could be attributed to several factors usually explained through window periods- and occult HBV infection. There are two window period in HBV infection when the detection of HBsAg could be missed. The period between infection and appearance of HBsAg and the period that elapses between the disappearance of surface antigen (HBsAg) from serum and the appearance of HBsAb (anti-HBs). 16 Seronegative occult HBV infection occurs in chronic carriers and is characterized by very low HBV DNA load in plasma (<200 IU/mL). Individuals with this infection could also escape being detected by the HBsAg assays even with appropriate capture and detection antibodies. 17, 18 This is because the concentration of HBsAg in plasma may be below the threshold for assay detection of the kit used. Some chronically infected persons actually have fluctuating HBV viremia. The lack of detectable HBsAg could also be attributed to replication deficiencies and viral genome variability particularly in the S and pre-S gene. Antigenicity and the immunogenicity of the HBsAg are altered by the viral genome mutation detection of HBsAg.^19^ Presence of immune complexes could also as well lead to decreased reactivity in HBsAg detection assays^20^ with NAT positivity in seronegative samples. The prevalence rate of NAT positive result for HBV DNA is relatively higher in other studies carried out in Nigeria than the prevalence reported in our study. This may be attributed to the screening algorithm in which only one serological assay type was used. In a center, with HBV NAT prevalence of 8.7%, point of care testing (rapid test) was used as a sole laboratory test.^19^ Another study that used only one ELISA assay, gave an HBV NAT prevalence of 5.4%.^20^ The lower prevalence in our study may be due to the complementary value of two different serological assay methods employed.

HBV NAT combines the ability to significantly reduce the window period and to detect occult HBV carriage. The HBV DNA positive blood donor identified in this study had a very high HBV DNA concentration of 768 000 IU/ml. The implication of HBV DNA in blood units to recipients is dependent on a number of factors. These include the anti-Bs titre and /or anti-HBc (if present), HBV DNA level, the type of blood component transfused and the recipient immune status^20^ The presence and titre of antiHBS and antiHBc are critical to transmission of HBV from a donor with OBI to a recipient. While anti-HBc is used to check for both a previous exposure to HBV and OBI, anti-HBs is indicative of viral persistence in otherwise resolved infections. OBI carriers (individuals with low HBV DNA copies) with high anti-HBs levels are unlikely to transmit the infection, whereas those with ‘anti-HBc only’ might transmit the infection. Conversely, the presence of anti-HBc and anti-HBs antibodies in blood donors would reduce the risk of HBV infection^20^. Whereas NAT helps to pick early HBV infections (reducing the window period to about 20 days, or less), anti-HBc picks late infections. therefore NAT and serological testing are often used in parallel.^21^ Interpretation of the result of the donor may be difficult even if anti-HBc and anti-HBs assays were done because both anti-HBc and anti-HBs may be absent in donations during an early viraemic window period.^22^, ^23^

The viral load detected in the donor carries a significant public health implication and poses threat to blood safety. In the presence of high viral load, the protective level of anti-HBs is debatable.^21^ The viral load provide information regarding infectivity or indirectly help with classification of the sample. Window period and escape mutants samples tend to have higher viral load than OBIs (<100 IU/ml).^21^ The number of HBV DNA copies present in blood products is one of the factors that determine clinical outcome when HBV DNA contaminated blood unit is transfused. Although immune status of recipient affects the relationship between infectivity and viral load in blood components, the use of whole blood rather than red cell concentrate in our anaemic patients should seriously be reconsidered to reduce viral dose exposure.^16^

The HBV genotype E observed in the HBsAg negative HBV DNA positive blood donor is the most prevalent in Nigeria.^24^ The role of possible impairment in the recognition of genotypes E by the HBsAg assay has been suggested by another author.^17^ Given the concern on the infectious risk, the most appropriate and effective strategy to minimize the risk of HBV infection among blood recipients should be sought. Use of NAT in addition to the serological screening is worth considering. Assay kit with significantly low antigen end-point analytical sensitivity which is also good in detecting genotype E should be sought in the event of inability to carry out NAT. If a blood bank cannot afford NAT because of the prohibitive cost, the regional laboratory could be set up to receive blood collected from a region for mini-pool NAT assay. Although there are arguments on the value of individual NAT assay (ID-NAT) over mini-pool (MP) NAT, MP-NAT has been proved to be more effective than HBsAg testing and capable of excluding infected donors with OBI and in the window periods.^25^ Hepatitis B vaccination is already widespread in the country to “At-risk professional individuals” and incorporated into the childhood immunization programs to reduce HBV in the community. Another approach to decreasing the risk of TT-HBV is the introduction of pathogen reduction technologies (PRT). This approach is not 100% effective against infectious agents present in high loads. Few reports have demonstrated that implementation of PRTs in resource-limited settings is feasible.^26^ Therefore, pathogen reduction may be considered for fresh frozen plasma and platelet concentrate to reduce infectious risk of HBV infected donation. It has not been approved for whole blood and red cell concentrate which is the major product supplied to patients in our hospitals.^5^ The strength of this study is that the study reflects real life procedure involved in producing blood units for patients in the blood bank.

## Limitation of our study

In view of the small sample size, our results thus may not reveal the overall prevalence of HBV in the screened donor units. Despite the limitations of this study, it shows that the interception of the transmission of HBV through high standard lab techniques is required. The use of two assays for HBsAg serological screening of blood donors does not completely eliminate the threat of HBV in blood units in high endemic region. Addressing the transmission of HBV through blood transfusion will significantly contribute to the prevention and control of HBV infection in the population. Further study with larger sample size and assessment of the anti-HBs titre with anti-HBc are recommended.

## Conclusion

Despite the use of two different types of tests to screen for HBsAg in blood donors, there is still a substantial risk for posttransfusion HBV infection. The inclusion of NAT even as mini-pool or pathogen reduction technologies will be cost-effective and rational given the high prevalence of HBV DNA in seronegative blood units.
